# A Review of the Phytochemistry and Bioactivity of Clover Honeys (*Trifolium* spp.)

**DOI:** 10.3390/foods11131901

**Published:** 2022-06-27

**Authors:** Sharmin Sultana, Kevin Foster, Lee Yong Lim, Katherine Hammer, Cornelia Locher

**Affiliations:** 1 Division of Pharmacy, School of Allied Health, University of Western Australia, Perth 6009, Australia; sharmin.sultana@research.uwa.edu.au (S.S.); lee.lim@uwa.edu.au (L.Y.L.); 2 UWA School of Agriculture and Environment, University of Western Australia, Perth 6009, Australia; kevin.foster@uwa.edu.au; 3 Cooperative Research Centre for Honey Bee Products Limited, 128 Yanchep Beach Road, Perth 6035, Australia; katherine.hammer@uwa.edu.au; 4 School of Biomedical Sciences, University of Western Australia, Perth 6009, Australia

**Keywords:** clover honey, phytochemistry, bioactivity, isoflavonoids, legumes

## Abstract

This review covers a comprehensive overview of the phytoconstituents and bioactivities reported to date for clover honeys produced from various *Trifolium* spp. against the backdrop of a more general discussion of the chemistry and bioactivity of these important agricultural species. While research into the phytochemical composition of various honeys and their associated bioactivities is growing, this review demonstrates that the literature to date has seen only a limited number of studies on clover honeys. Surprisingly, there appear to be no comparative data on the concentration of flavonoids in general or isoflavonoids specifically in different clover honeys, although the latter have been identified as a main group of bioactive compounds in red clover plants. Based on the findings of this review, the presence of phytoestrogenic isoflavonoids (e.g., formononetin, biochanin A, genistein, daidzein, glycitein) in clover plants and, by extension, in clover honeys should be further investigated, specifically of clover species outside the three popular perennial clovers (red, white and alsike clovers) to exploit new opportunities of potential benefit to both the pharmaceutical and apiculture industries.

## 1. Introduction

European honey bees (*Apis mellifera*) generate a variety of natural products of nutritional and in many instances also of medicinal value such as honey, but also bee pollen, propolis and royal jelly. Honey is a sweet and flavorful natural product that has been consumed for centuries for its high nutritive value but has also been used for thousands of years as a medicinal agent, with the earliest records of medicinal use dating back to the Ancient Egyptian era [1]. Honey products promote many beneficial human responses due to their bioactive constituents, including antimicrobial, antiviral, antioxidant, anti-inflammatory and antidiabetic effects [2,3]. Honey is a highly complex mixture of at least 200 phytochemicals with its composition being strongly influenced by multiple factors, including its botanical and geographical origin, the bee species involved in its production, its age, method of storage and processing [4,5].

Clover honeys, considered a premium product worldwide, are a unique type of honey produced from various species of the genus *Trifolium* [6]. Clovers are commonly a predominant species in multifloral honeys, indicating bee preference for these crops in an agricultural setting [7]. It is therefore no surprise that species such as *T. incarnatum* L. (crimson clover), *T. pratense* L. (red clover) and *T. repens* L. (white clover), but also *T. michelianum* Savi. (balansa clover), *T. vesiculosum* L. (arrowleaf clover) and *T. fragiferum* L. (strawberry clover) are all considered as being a relatively high value crop for honeybees [8].

Monofloral bioactive honeys are highly sought after and priced accordingly [9], as seen in the growing global demand for specialist pharmaceutical honeys such as the Manuka (*Leptospermum scoparium*) honey. There is also a strong impetus to develop various legume honeys into medicinal honeys [10]. Based on the plants’ well established phytochemical profile, clover honeys could indeed be a potential source of bioactive honeys. In this context, integrated studies on the chemical composition and biological activity of clover honeys will be very important in establishing their potential therapeutic applications.

In support of this call for more research on clover honeys, this review focuses on the physicochemical characteristics, key phytochemical constituents and different bioactivities reported for clover honeys of different botanical origins. Against the backdrop of a brief review of honey chemistry in general as well as a short discussion of the clover plant genus, a systematic literature review is presented on the body of peer-reviewed scientific research on clovers and their interaction with European honeybees. To compile this review, the data bases PubMed^®^, ScienceDirect™ and Google Scholar were searched between January 2022 and February 2022 using different combinations of the following terms: ‘clover’, ‘clover honey’, ‘chemistry of clover honey’, ‘bioactivity of clover honey’ “*Apis mellifera*”, and ‘European honeybee’. Multiple clover species names were identified during the search and confirmed through consultation with specialists in legume research at The University of Western Australia and the Department of Primary Industries and Regional Development (DPIRD). Titles and abstracts obtained in this primary literature search were considered to select articles for full review, and the reference sections from each article was subsequently searched for related publications of interest.

## 2. The Chemistry of Honey

Honey is a highly concentrated complex mixture of mainly sugars (75–85%), water (13–20%) and a small fraction of non-sugar constituents (approx. 3%) [4,5,8,11]. The principal carbohydrate constituents of honey are fructose (approx. 33 to 38%) and glucose (approx. 28 to 31%), which represent 85–95% of total sugars that are readily absorbed in the gastrointestinal tract [12,13]. Other sugars include disaccharides, such as maltose, sucrose, isomaltose, turanose, nigerose, melibiose, panose, maltotriose and melezitose, as well as 4 to 5% fructooligosaccharides, which can serve as prebiotic agents [13,14]. Honey’s non-sugar constituents, though only present in relatively small quantities, are considered to be vital in influencing not only its organoleptic and physicochemical characteristics but also its bioactivity profile [15]. Organic acids constitute approximately 0.6% of honey and include, amongst others, gluconic acid, which is a by-product of enzymatic digestion of glucose. The organic acids are responsible for the acidity of honey, resulting typically in a pH range of 3–5, and contribute largely to its characteristic taste [16]. The concentration of mineral compounds in honey ranges from 0.1% to 1.0%. Potassium is the major metal, followed by calcium, magnesium, sodium, sulphur and phosphorus. Trace elements include iron, copper, zinc and manganese [17,18,19]. Nitrogenous compounds, vitamins C and B_1_ (thiamine), and B_2_ complex vitamins like riboflavin, nicotinic acid, vitamin B_6_ and pantothenic acid are also present [16]. Furthermore, honey contains proteins, but only in minute quantities (0.1–0.5%) [20,21]. Plant pollen, a natural contaminant found in honey as a result of bees’ foraging activities, contributes significantly to honey’s overall protein content [2,7,9]. In addition, according to a recent report, specific protein quantities in honey also differ depending on the species of honeybee producing the honey [22], since honey’s protein content is also related to the presence of a variety of bee enzymes. The main enzymes found in honeybee derived honeys are glucose oxidase, invertase (saccharase), diastase (amylase) and catalase. They play an important role in the composition and, in parts, also the bioactivity of honey [16]. Glucose oxidase, for example, is important as it produces hydrogen peroxide, which provides the honey with antimicrobial properties, as is gluconic acid, which aids calcium absorption in humans. Invertase, on the other hand, converts sucrose to fructose and glucose, while dextrin and maltose are produced from long starch chains by the activity of amylase. Catalase generates oxygen and water from hydrogen peroxide and its presence thus has a negative effect on a honey’s hydrogen peroxide-related antibacterial activity [23].

## 3. Clover Honeys

Clover is the common name used for plants of the genus *Trifolium*, which includes both annual and perennial species. They are short leguminous herbs with trifoliate leaves and compact flower heads and include numerous important crops for pasture animal forage, which are also alluring to bees. Clover plants belong to the large and economically important family of Fabaceae or Leguminosae, one of the largest families of flowering plants [24], and the third biggest land-based plant family, comprising 730 genera and over 19,400 species [25]. Of all the plants that humans currently use as a food source, perhaps only grasses (Gramineae) exceed the importance of legumes [26]. Many of the *Trifolium* species are cultivated extensively as fodder plants and, like other members of the legume family, they naturally add nitrogen to the soil, a process known as biological nitrogen-fixation. Legumes also add other value in terms of weed, insect, and pathogen control and in increasing soil organic matter when rotated with crops in farming systems [27].

Research has demonstrated that *Trifolium* spp. exhibit a range of biological effects, including antioxidant, anticestodal, anti-inflammatory, cytotoxic, cytostatic and estrogenic activities. They are thus popular in some traditional medicinal applications and used, for instance, as chemoprotective agents against cancers and cardiovascular diseases [28,29,30]. In recent decades, several studies have established the nutritious value and bioactive compounds in various members of the Fabaceae, including red clover [31,32,33,34]. A range of isoflavonoids alongside volatile and fatty oils, vitamin C and carotene have been identified from the flower heads and leaves of red clover. Being rich in isoflavones, red clover has been found to be able to prevent several conditions and diseases [34,35,36]. Secondary metabolites isolated from its flower heads have, for example, been demonstrated to possess beneficial effects in the management of menopausal disorders, cardiovascular diseases and several cancers [37,38,39]. Menopause, an intermediate phase from a reproductive to a non-reproductive period in a woman’s life, is characterised by a decrease in estrogen, which triggers a range of uncomfortable symptoms including hot flushes, night sweats, cognitive change, anxiety, depression, sleep disturbances and vaginal dryness [40,41,42], which can significantly affect quality of life. The use of red clover in the treatment of menopausal symptoms is popular, based on the general premise that isoflavones (e.g., formononetin, genistein, daidzein, and biochanin A) will act as a natural estrogen replacement in menopausal women and thus alleviate climacteric symptoms [31,39]. Moreover, phytoestrogens and other flavonoids found in red clover are effective antioxidants and may have tyrosine kinase inhibitory activity [42].

Clovers are a prolific source of pasture honeys, which are characterised by a strong nectar aroma and a light nectar flavour. The aerial clovers also often have uncovered anthers and stigmas in the flowers, offering a floral morphology that is functionally well suited to pollination by honey bees [43]. Inflorescences are usually branched racemes or panicles, providing a high number of flowers per planting area that attracts pollinators [44]. The most important clovers for honey production to date are red clover, white or Dutch clover, alsike clover (*T. hybridum*) and crimson clover (*T. incarnatum*). The honeys harvested from these species are mainly appreciated for their distinct and complex flavour profiles.

Although some clover honeys are already produced commercially, very little is known about their phytochemical profile. For the vast majority of the about 100 different *Trifolium* species in the Australian Pastures Genebank, for example, no data is yet available on the honeys that might be produced from them, such as their phytochemical characteristics and bioactivities. To address this gap in knowledge, in this study we present information pertaining to the phytochemistry of different clovers followed by a review of the scarce literature on clover honeys.

### 3.1. Phytochemical Composition and Bioactivity of Clover Plants

Kaurinovic et al. [42] investigated the presence of phenolic compounds in *T. pratense* leaf extracts and found, not surprisingly, that the extraction solvent played an important role, with the ethyl acetate extracts yielding the highest total phenolic content (0.43 ± 0.01 mg gallic acid equivalents (GAE)/g dry extract (d.e.). A significant amount of phenolic compounds was also observed in the aqueous extract (0.34 ± 0.03 mg GAE/g d.e.) whereas the amounts of total phenolics found in the diethyl ether and n-butanol extracts were lower, at 0.22 ± 0.03 mg GAE/g d.e. and 0.21 ± 0.03 mg GAE/g d.e., respectively. A considerable total flavonoids content was also determined in the aqueous (15.13 ± 0.05 µg rutin equivalents (RE)/g d.e.) and ethyl acetate (15.23 ± 0.01 µg RE/g d.e.) extracts [37], less so in the n-butanol (11.87 ± 0.03 µg RE/g d.e.) extracts, and the smallest quantities of total flavonoids were recovered from the diethyl ether (11.78 ± 0.04 µg RE/g d.e.) and chloroform extracts (9.24 ± 0.03 µg RE/g d.e) [42]. Next to the chosen extraction solvent, the presence and concentration of secondary metabolites like flavonoids mainly depend on the plant’s development stage, its genotype, as well as the plant part itself, so concentrations of bioactive constituents vary considerably between stems, leaves and flowers [31,45,46].

**Figure 1:**
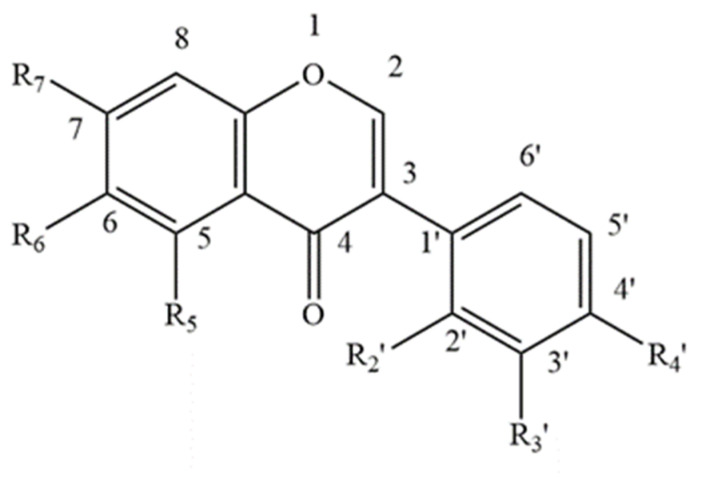
Basic Isoflavone structure.

**Figure 2:**
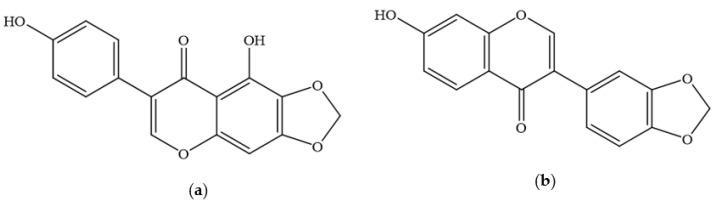
Structure of Irilone (**a**) and Pseudobaptigenin (**b**).

**Figure 3:**
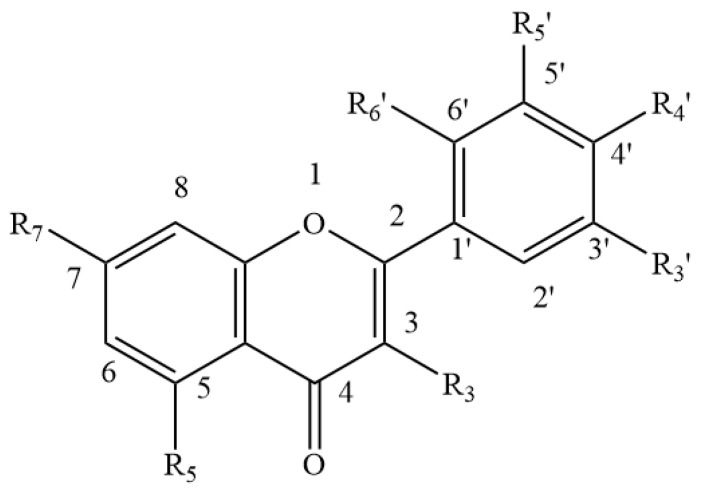
Basic Flavonoid structure.

**Figure 4:**
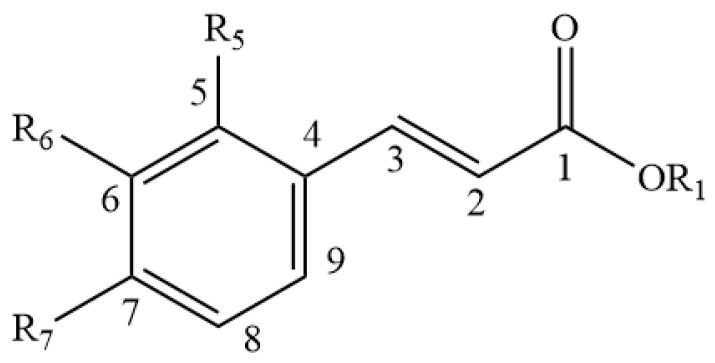
Basic structure of Hydroxycinnamic acid and its derivatives.

**Figure 5:**
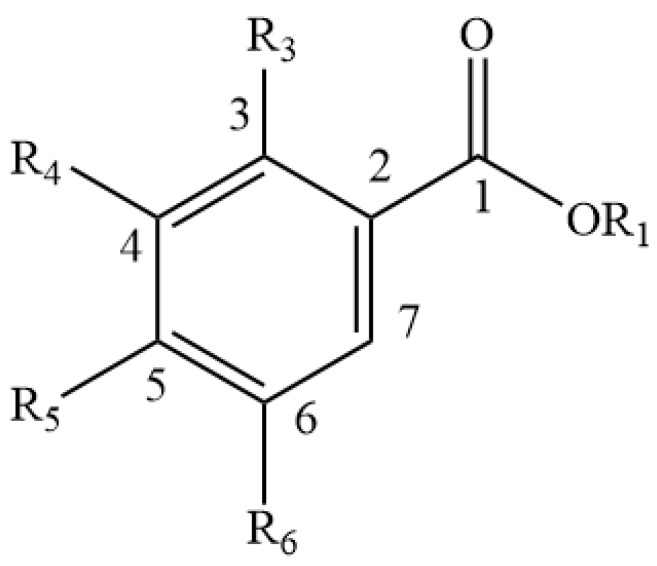
Basic structure of Hydroxybenzoic acid and its derivatives.

**Figure 6:**
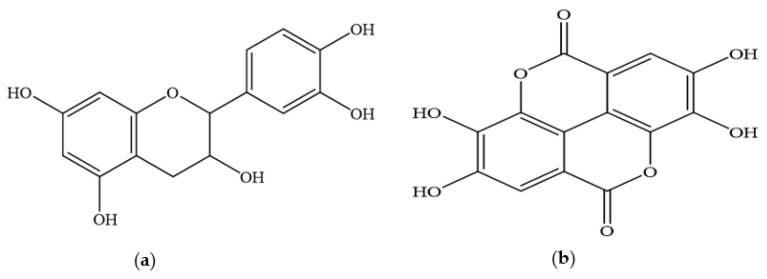
Structure of Catechin (**a**) and Ellagic acid (**b**).

Approximately 40 different isoflavones have to date been reported from red clover plants [47,48], the main ones being biochanin A and formononetin, along with lower concentrations of daidzein, glycitein and genistein (Table 1 and Figure 1) [49,50,51]. Other isoflavones (Table 1 and Figure 1 and Figure 2) found in the leaves of red clover plants include calycosin, prunetin, afrormosin, texasin, irilin B, irilone and pseudobaptigenin, which were identified through several studies [52,53,54] alongside other flavonoids such as quercetin and kaempferol (Table 2, Figure 3). Furthermore, red clover is characterised by the presence of other phenolics, in particular phenolic acids such as caffeic, rosmarinic and chlorogenic acid (Table 3 and Figure 4) [42]. Other clover plants, for example alsike clover, have also been found to contain phenolic constituents along with other compounds. High Performance Liquid Chromatography (HPLC) analyses of ethanolic extracts of alsike clover flowers have identified chlorogenic acid, cryptochlorogenic acid, *p*-coumaric acid, quercetin, gallic acid, syringic acid and catechin (Table 2, Table 3 and Table 4 and Figure 3, Figure 4, Figure 5 and Figure 6) [55]. Quercetin was the major phenolic compound detected and found to be responsible for several bioactivities (e.g., antioxidant, antimicrobial activity etc.) in alsike clover flowers [55,56]. Their antioxidant activity was confirmed in the 2,2-diphenyl-1-picrylhydrazyl (DPPH) assay, where DPPH is used as a stable free radical to capture the antiradical activity of honey and a dose-dependent antimicrobial activity against bacteria (*E. coli*, *Salmonella typhi*, *Bacillus aureus*, *S. aureus*), yeast (*C. albicans*) and fungi (*Aspergillus flavus*) was also reported [55].

**Figure 7:**
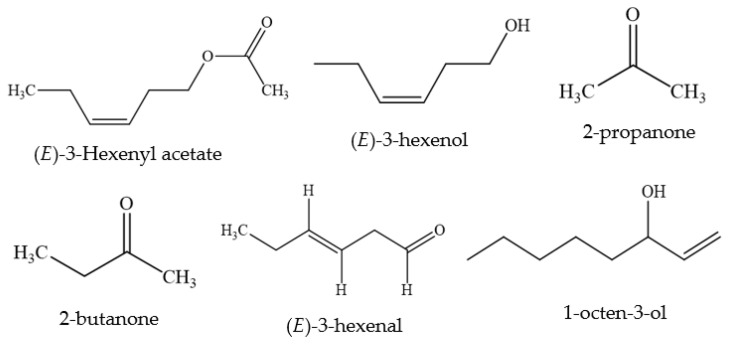
Volatile components reported in Clover plants.

In addition to some phenolic constituents, volatile components present in different clover species have also been determined. A study, carried out by Massei et al. [57], identified volatile components (Table 5 and Figure 7) in red clover, strawberry clover and white clover. (*E*)-3-Hexenyl acetate and (*E*)-3-hexenol were present in all three species, but in substantially larger amounts in white clover. Two major volatiles of white clover, 2-propanone and 2-butanone, were found only in this species. These two key volatiles were found to be released in significant quantities from the oil extract alongside smaller amounts of (*E*)-3-hexenal, 1-octen-3-ol and (*E*)-3-hexenyl acetate as well as (*E*)-3-hexenol, which was produced by damaging the growing plant [58]. It has also been found that white clover contains cyanogenic glycosides, which liberate toxic levels of hydrogen cyanide (HCN) and so provide an immediate chemical defense response to herbivores and pathogens causing damage [30].

In summary, the review of the literature has unveiled several studies reporting biological activities for red and alsike clovers [28,29,30], though other *Trifolium* spp. are also used as medicinal plants. Red clover extracts are, for example, taken internally for the treatment of skin complaints (especially eczema and psoriasis), cancers of the breast, ovaries and lymphatic system, chronic degenerative diseases, gout, whopping cough and dry coughs [59]. Extracts of red clover are also becoming increasingly popular for the treatment of menopausal symptoms [60,61,62]. Furthermore, phytoestrogens present in red clover are also effective antioxidants and may have tyrosine kinase inhibitory activity. The antioxidant properties of genistein and other phytoestrogens have, for example, been demonstrated in vitro in several models such as protection from phorbol ester-induced singlet oxygen or peroxide formation and particularly from UV-radiation induced oxidative damage to DNA [63,64,65]. In animal models, particularly in mice, dietary genistein has been shown to stimulate the endogenous antioxidants superoxide dismutase (SOD), glutathione peroxidase (GSHPx) and glutathione reductase (GSHR) as well as glutathione S-transferase, with the antioxidant effects (i.e., protection against free radical-induced cell damage by metabolizing oxidative toxic intermediates) seen mainly in the small intestine and the skin [66,67].

### 3.2. Phytochemical Composition of Clover Honey

There is an increasingly positive market trend for honey products with medicinal benefits within the retail and pharmaceutical industry following in particular the success of the highly antibacterial New Zealand Manuka honey. While research into the phytochemical composition of various honeys and their associated bioactivities is growing, the literature to date has seen only a limited number of studies on clover honeys. Surprisingly, there appear to be no comparative data on the concentration of flavonoids in general or isoflavonoids specifically in different clover honeys although the latter have been identified as the main group of bioactive compounds in red clover plants. The presence of phenolic acids and flavonoid compounds, specifically isoflavones, in clover honeys is, however, to be expected, given that honeybees collect nectar from clover flowers that have been shown to contain these potentially bioactive compounds [68,69,70,71].

**Figure 8:**
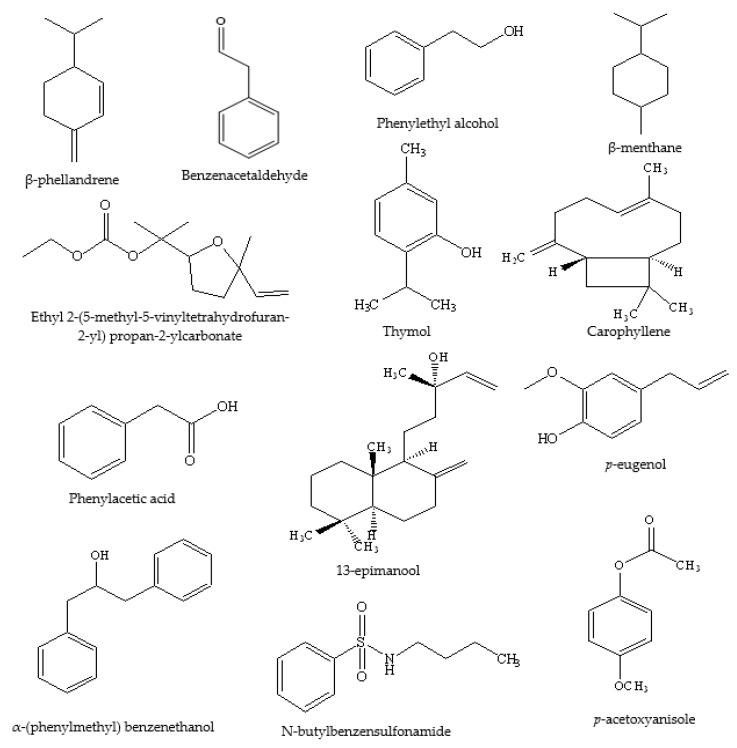
Volatile components found in Clover honey.

**Figure 9:**
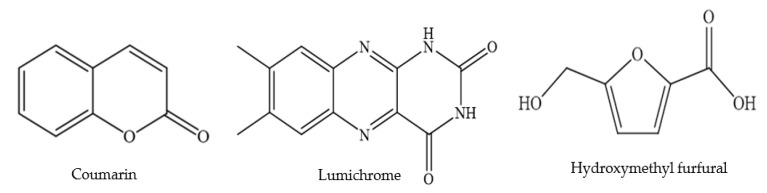
Miscellaneous compounds.

With respect to other phenolic compounds, high performance liquid chromatography-pulsed amperometric detection (HPLC-PAD) analysis of Polish sweet clover honey (*Melilotus officinalis* L.), for example, has indicated the presence of 15 phenolic compounds, nine of them phenolic acids (Table 3 and Table 4 and Figure 4, Figure 5 and Figure 6) (caffeic, rosmarinic, *p*-coumaric, ferulic, cinnamic, gallic, *p*-hydroxybenzoic, m-hydroxybenzoic, ellagic) and six flavonoids (Table 1 and Table 2 and Figure 1, Figure 3 and Figure 6) (genistein, quercetin, morin, myricetin, pinocembrin, (+)-catechin) [72]. Among these 15 phenolic compounds, the amounts of gallic acid (23.23 ± 4.52 mg/100 g honey) and (+)-catechin (26.79 ± 2.99 mg/100 g honey) present were considered significant [72]. Moreover, gas chromatography-mass spectrometry (GC-MS) analysis of dichloromethane extracts, obtained after steam distillation, Soxhlet extraction, and ultrasonic solvent extraction (USE) of Polish sweet yellow clover honey led to the identification of a range of volatile compounds (Table 5 and Figure 8). When using the steam distillation technique, β-phellandrene (2.17%), benzenacetaldehyde (5.84%), β-menthane (2.23%), thymol (2.03%) and ethyl 2-(5-methyl-5-vinyltetrahydrofuran-2-yl) propan-2-ylcarbonate (2.84%) were detected. Soxhlet extraction yielded carophyllene (11.23%), 13-epimanool (1.45%), phenylacetic acid (5.35%) and *p*-eugenol (6.54%), and phenylethyl alcohol (2.03%), while the predominant compounds isolated using USE were phenylacetic acid (25.54%), α-(phenylmethyl) benzenethanol (6.44%) and *p*-acetoxyanisole (2.89%). In the case of solid phase extraction (SPE), two major compounds were identified, phenylacetic acid (13.04%) and N-butylbenzensulfonamide (10.94%) [72]. Coumarin and lumichrome (Table 5 and Figure 9), a breakdown product of riboflavin, which forms in the presence of light in neutral or acidic solutions, were also isolated from Polish sweet yellow clover honeys using the SPE method. The authors suggest that the presence of coumarin in sweet yellow clover honey might be used as a marker to authenticate the honey’s floral source alongside the presence of lumichrome and a high concentration of phenylacetic acid [68,72].

*p*-Coumaric acid was detected as the main phenolic component in the ethanol extract of alsike clover honey [55]. It is responsible for the honey’s characteristic sweet odour and has been linked to some health-promoting properties, including sedative, spasmolytic, anti-inflammatory and antithrombotic activities. It is proposed to strengthen lymphatic vessels, stimulate blood and lymph flow and for that reason alsike clover honey is suggested to be used in the prophylaxis and treatment of lymphedema as well as chronic venous disease (CVD) [69,70,71]. Similar to the flower extract of alsike clover, the HPLC analysis of the honey’s ethanolic extract also revealed the presence of quercetin, chlorogenic acid, cryptochlorogenic acid, *p*-coumaric acid, gallic acid, syringic acid and catechin [55].

**Figure 10:**
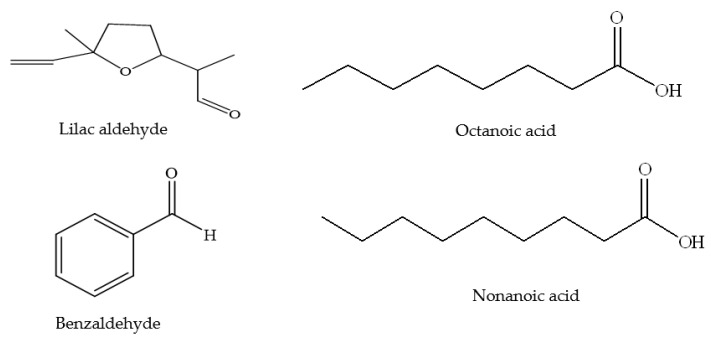
Volatile constituents of red clover honey.

Volatile constituents of red clover honey were investigated by Jerkovic et al. [38]. They identified 12 compounds (Table 5 and Figure 10) by headspace solid-phase micro-extraction (HS-SPME) [38]. The dominant compounds were lilac aldehyde isomers ranging individually from 7.6% to 21.4%. Other major compounds were benzene derivatives such as phenylacetaldehyde (10.1–31.2%) and benzaldehyde (7.0–15.7%) [38]. The authors concluded that an abundance of lilac aldehydes together with phenylacetaldehyde and benzaldehyde can be considered as the typical volatile profile of red clover honey [38]. These volatile constituents have frequently been found in honey and have been demonstrated to originate from phenylalanine as a precursor compound [73]. Two lower molecular weight aliphatic acids were also found during the headspace analysis of red clover, namely octanoic acid (0.7–2.9%) and nonanoic acid.

**Figure 11:**
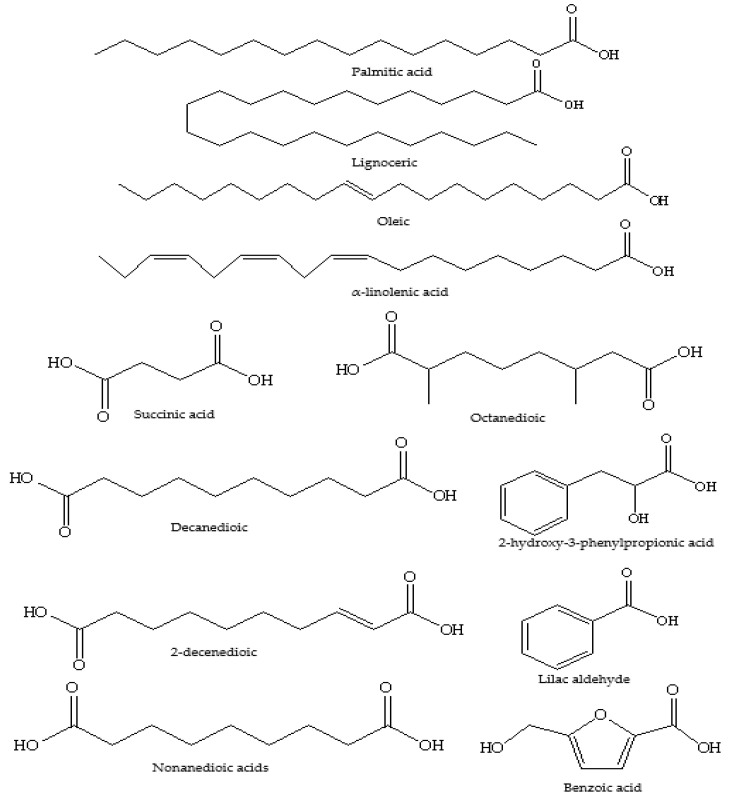
Structure of compounds reported in white clover honey.

A range of acidic constituents (Table 5 and Figure 11) have been isolated and identified from extracts of aqueous solutions of white clover honey by Tan et al. [74]. Next to hydrocarbons (C21–C33), they reported the presence of a variety of straight-chain monoacidic compounds (C8–C28). Palmitic, lignoceric, oleic and α-linolenic acid were dominant in content alongside diacid (succinic acid, octanedioic, decanedioic, 2-decenedioic, nonanedioic acids) and aromatic compounds (2-hydroxy-3-phenylpropionic acid, (*E*)-cinnamic acid, phenylacetic acid, and benzoic acid) [74].

Minerals and trace elements in clover honeys have also been reported. Though not a legume per se, a study conducted by Zaghloul et al. [75] on ‘Brazilian Clover’ (*Richardsonia brasiliensis*) honey reported that potassium was the most abundant element (306.600 mg/100 g). In addition, the honey was also found to contain other minerals such as Ca, Na, Mg, Zn, Cu, Fe, Mn and Pb [75].

Hydroxymethyl furfural (HMF) (Table 5 and Figure 9) is an important indicator in evaluating the freshness of honey [76,77], as it can be formed as a heat-triggered degradation product of monosaccharides. Thus, the formation of HMF is affected by many factors such as a honey’s fructose content, the type of other sugars present, its fructose to glucose ratio, storage and other treatment temperatures, pH, the age of the honey and ultimately its floral source [77,78]. A comparative chemical analysis of Brazilian pepper, clover and citrus honeys was conducted by Zaghloul et al. [75]. Data revealed that clover honey contained high levels of HMF (77.13 mg/kg), while Citrus honey had the lowest content (10.73 mg/kg) [75]. It is not clear, however, from the findings of this study, if higher HMF levels are an inherent feature of clover honeys or if these results were triggered by specific handling, processing and storage conditions. Since HMF is associated with potentially negative health outcomes [79,80], its presence in honey is limited by the Codex Alimentarius to 40 mg/kg [72,81]. However, in the case of honeys of declared origin from countries or regions with tropical ambient temperatures, and blends of these honeys, the HMF content shall not exceed 80 mg/kg [72,81]. The collation of more data on typical HMF concentrations in different clover honeys should therefore be a focus of future research.

### 3.3. Bioactivity of Clover Honeys

A review of the literature on the chemistry of clover honeys has only been able to identify limited studies that reported on the phytochemical constituents of these honeys. Similarly, to date, only a few studies have investigated the bioactivities (Table 6) of clover honeys. These studies are briefly discussed in the following section.

Without focusing on specific individual compounds, a study conducted by Smetanska et al. [82] revealed that the total phenolic content in Egyptian clover honey (*T. alexandrinum* L.), determined using the Folin Ciocalteau method, varied from 18.574 mg to 53.314 mg gallic acid equivalent/g honey. Similarly, the authors also investigated the total flavonoid content in Egyptian clover honey by a complexation reaction with AlCl_3_ and found it to be 26.604 mg catechin/kg honey [82]. By extension, these unspecific quantifications can be seen as a broad assessment of the antioxidant activity of Egyptian clover honey.

More specific bioactivity studies to date have mainly investigated the antioxidant and antimicrobial activities of honeys. In the case of red clover honeys, they have been found in two studies to exhibit antioxidant activity to different degrees [38,82]. In one, red clover honey showed moderate radical scavenging activity in the DPPH assay, where DPPH (2,2-diphenyl-1-picrylhydrazyl) is used as a stable free radical to capture the antiradical activity of honey [38]. In another study that employed the ferric-reducing antioxidant power (FRAP) assay, another widely applied method for the determination of total antioxidant activity of natural products including honeys [33], Jerkovic et al. [38] reported a weak antioxidant activity for red clover honey.

Honey has also been known since ancient times as a therapy for infected wounds. In clinical practice today honey is used as a topical wound treatment, especially for those wounds that do not respond to conventional therapies, such as diabetic ulcers [83,84]. Diabetic foot infection, most commonly with *Proteus mirabilis*, is a serious complication of diabetes that can lead to amputation of the lower extremities. The infection is problematic to treat due to the high resistance to antibiotics and biofilm formation. Egyptian clover honey was found to exert antibacterial activity against *P. mirabilis* at an aqueous concentration of 40% (*w*/*v*) and to completely inhibit the swarming motility of *P. mirabilis* at 20% (*w*/*v*) concentration [85]. Its activity was deemed to be bactericidal since its Minimum Bactericidal Concentration (MBC)/Minimum Inhibitory Concentration (MIC) was equivalent to 1 [85]. Moreover, *P. mirabilis* was also shown to be inhibited by Egyptian clover honey at a concentration of 35% (*w*/*v*) [86], which confirms the antimicrobial activity of this honey as reported by others [85]. Translating these findings into practice, the application of Egyptian clover honey dressings in a clinical trial involving 30 patients with infected diabetic foot ulcers was found to result in the complete healing of 43.3% of ulcers, significant reduction in ulcer size for 43.3% of patients, and a lowering of the bacterial burden in all ulcers after the first week of therapy [87].

Furthermore, the antimicrobial effects of alsike clover honey were investigated by Roby et al. [55], who reported activity against bacteria (*Escherichia coli*, *Salmonella typhi*, *Bacillus cereus*, *Staphylococcus aureus*), one strain of yeast (*Candida albicans*), and one strain of fungi (*Aspergillus flavus*). In a separate study conducted by Khairy et al. [86], clover honey (regrettably the specific botanical *Trifolium* species was not stipulated) demonstrated also good antibacterial activity against *Pseudomonas aeruginosa* and *S. aureus*. Similarly, Egyptian clover honey at an aqueous concentration of 20.3% (*w*/*v*) inhibited the growth of *S. aureus*, *P. aeruginosa* and *Klebsiella pneumoniae* [88].

Outside the medical field, clover honeys have also been investigated for potential applications. Corrosion is a natural process commonly defined as the deterioration of metal surfaces caused by reaction with the surrounding environment. A common method for corrosion control and prevention is to apply corrosion inhibitors. Most commercial corrosion inhibitors are expensive and are health and environmental hazards. In recent years, the focus has been directed towards exploring naturally occurring materials and their extracts to produce cheaper, non-toxic, and “green” corrosion inhibitors. Due to its biodegradability, eco-friendliness and low cost, honey with its antioxidant activity may fulfil the requirements of a non-toxic natural corrosion inhibitor. Several studies have already confirmed the potential application of honey as an inhibitor of corrosion in different metals and alloys [89,90,91,92,93]. Aluminium and its alloys are the most used materials in the food, pharmaceutical and cosmetics industries. In one study, the anticorrosive properties of red clover honey on aluminium AA 2017A alloy in NaCl solution (0.1 mol L^−1^), as determined by potentiodynamic polarization and potentiostatic pulse measurements, was found to be significant, indicating its potential as a new “green” corrosion inhibitor [38].

## 4. Conclusions

This comprehensive review presents the physicochemical characteristics, phytochemical constituents as well as bioactivities of clover honeys and clover plants around the globe. The literature review found numerous articles on red, white and alsike clovers, which are perennials (or short-lived perennials) and are traditionally grown in the high-rainfall regions of Europe and North America. This is not surprising, as the red and white clovers are the most widely cultivated perennial species used as fodder plants in the world. There are, however, numerous other aerial annual *Trifolium* species adapted to a broad range of soil types and rainfall zones that are not adequately covered by the current literature. These species have remained largely unassessed as pollen or nectar sources for the apicultural industry, and their bioactivity is underexplored as potential sources of new natural medicines.

*Trifolium* spp. are known to be a potent natural source of isoflavonoids used in traditional medicine to treat a number of health disorders. Red clover, for instance, has gained much popularity due to research into its use for the treatment for menopausal symptoms. The presence of unique isoflavonoids (e.g., formononetin, biochanin A, genistein, daidzein, glycetein) in clover plants and, by extension, in clover honeys might differentiate these in providing specific bioactivities (e.g., phytoestrogenic activity) that should be further investigated. Based on the findings of this comprehensive literature review, a more in-depth exploration is warranted, in particular of clover species outside the three popular perennial clovers (red, white and alsike clovers) to exploit new opportunities of potential benefit to both the pharmaceutical and apiculture industries.

## Figures and Tables

**Table 1:** Isoflavones reported in clover plants and honey (see Figure 1 for general structure).

Name	R5	R6	R7	R2′	R3′	R4′
Biochanin A	-OH	-H	-OH	-H	-H	-O-Me
Formononetin	-H	-H	-OH	-H	-H	-O-Me
Daidzein	-H	-H	-OH	-H	-H	-OH
Glycitein	-H	-O-Me	-OH	-H	-H	-OH
Genistein	-OH	-H	-OH	-H	-H	-OH
Calycosin	-H	-OH	-H	-H	-OH	-O-Me
Prunetin	-OH	-H	-O-Me	-H	-H	-OH
Afrormosin	-H	-O-Me	-OH	-H	-H	-O-Me
Texasin	-H	-O-Me	-O-Me	-H	-H	-O-Me
Irilin B	-OH	-O-Me	-OH	-OH	-H	-H
Irilone	N/A	N/A	N/A	N/A	N/A	N/A
Pseudobaptigenin	N/A	N/A	N/A	N/A	N/A	N/A

Legend: -H = hydride, -OH = hydroxide, -OMe = Methoxide.

**Table 2:** Flavonoids reported in clover plants and honey (see Figure 3 for general structure).

Name	R3	R5	R7	R3′	R4′	R5′	R6′
Quercetin	-OH	-OH	-OH	-H	-OH	-OH	-H
Kaempferol	-OH	-OH	-OH	-H	-OH	-H	-H
Morin	-OH	-OH	-H	-H	-OH	-H	-OH
Myricetin	-OH	-OH	-OH	-OH	-OH	-OH	-H
Pinocembrin	-H	-OH	-OH	-H	-H	-H	-H

Legend: -H = hydride, -OH = hydroxide.

**Table 3:** Hydroxycinnamic acid and its derivatives reported in clover plants and honey (see Figure 4 for general structure).

Name	OR1	R5	R6	R7
Caffeic acid	-H	-H	-OH	-OH
Rosmarinic acid	-3,4-DHPLA	-H	-OH	-OH
Chlorogenic acid	-QA (3)	-H	-OH	-OH
Cryptochlorogenic acid	-QA (4)	-H	-OH	-H
*p*-Coumaric acid	-H	-H	-H	-OH
Ferulic acid	-H	-H	-O-Me	-OH
Cinnamic acid	-H	-H	-H	-H

Legend: -H = hydride, -OH = hydroxide, -OMe = Methoxide, -QA = quinic acid.

**Table 4:** Hydroxybenzoic acid and its derivatives reported in clover plants and honey (see Figure 5 for general structure).

Name	OR1	R3	R4	R5	R6
Gallic acid	-H	-H	-OH	-OH	-OH
Syringic acid	-H	-H	-O-Me	-OH	-O-Me
*p*-Hydroxybenzoic acid	-H	-H	-H	-OH	-H
m-Hydroxybenzoic acid	-H	-H	-OH	-H	-H

Legend: -H = hydride, -OH = hydroxide, -OMe = Methoxide.

**Table 5:** Compounds reported in clover plants and clover honeys.

Category	Name	Quantity (Approximate)
Volatile components reported in clover plants	(*E*)-3-Hexenyl acetate	Not reported
	(*E*)-3-hexenol	Not reported
	2-propanone	Not reported
	2-butanone	Not reported
	(*E*)-3-hexenal	Not reported
	1-octen-3-ol	Not reported
Volatile components found in Clover honey	β-phellandrene	2.17%
	Benzenacetaldehyde	5.84%
	β-menthane	2.23%
	Thymol	2.03%
	Ethyl 2-(5-methyl-5-vinyltetrahydrofuran-2-yl) propan-2-ylcarbonate	2.84%
	Carophyllene	11.23%
	13-epimanool	1.45%
	Phenylacetic acid	5.35–25.5%
	*p*-eugenol	6.54%
	Phenylethyl alcohol	2.03%
	α-(phenylmethyl) benzenethanol	6.44%
	*p*-acetoxyanisole	2.89%
	N-butylbenzensulfonamide	10.94%
Volatile constituents found in red clover honey	Lilac aldehyde	7.6–21.4%
	Benzaldehyde	7.0–15.7%
	Octanoic acid	0.7–2.9%
	Nonanoic acid	Not reported
Compounds reported in white clover honey	Palmitic acid	Not reported
	Lignoceric	Not reported
	Oleic	Not reported
	α-linolenic acid	Not reported
	Succinic acid	Not reported
	Octanedioic	Not reported
	Decanedioic	Not reported
	2-decenedioic	32.1 µg/g
	Nonanedioic acids	Not reported
	2-hydroxy-3-phenylpropionic acid	2.5–66.9 µg/g
	Benzoic acid	Not reported
Miscellaneous compounds	Coumarin	0.05 to 0.88 mg/kg
	Lumichrome	1.85%
	Hydroxymethyl furfural	10.73–77.13 mg/kg

**Table 6:** Bioactivities of various clover honeys.

Bioactivity	Clover Honey Type
Antioxidant activity	Red clover; Egyptian clover
Antimicrobial activity	Red clover; Egyptian clover; Alsike clover
Wound healing activity	Egyptian clover
Anti-corrosive activity	Red clover

## Data Availability

Not applicable.
